# Mitogenome Analysis of Four Lamiinae Species (Coleoptera: Cerambycidae) and Gene Expression Responses by *Monochamus alternatus* When Infected with the Parasitic Nematode, *Bursaphelenchus mucronatus*

**DOI:** 10.3390/insects12050453

**Published:** 2021-05-14

**Authors:** Zi-Yi Zhang, Jia-Yin Guan, Yu-Rou Cao, Xin-Yi Dai, Kenneth B. Storey, Dan-Na Yu, Jia-Yong Zhang

**Affiliations:** 1 College of Chemistry and Life Science, Zhejiang Normal University, Jinhua 321004, China; cixi55@126.com (Z.-Y.Z.); a1345413239@163.com (J.-Y.G.); 13306760612@163.com (Y.-R.C.); kkr_xy@163.com (X.-Y.D.); 2 Department of Biology, Carleton University, Ottawa, ON K1S 5B6, Canada; KennethStorey@cunet.carleton.ca; 3 Key Lab of Wildlife Biotechnology, Conservation and Utilization of Zhejiang Province, Zhejiang Normal University, Jinhua 321004, China

**Keywords:** *Monochamus alternatus*, *Bursaphelenchus mucronatus*, Lamiinae, phylogeny, mitochondrial genome, mitochondrial gene expression

## Abstract

**Simple Summary:**

The longicorn beetle, *Monochamus alternatus*, is a major vector for the transmission of pine wilt disease, which is caused by a nematode pathogen, *Bursaphelenchus xylophilus* (or also possibly by *B. mucronatus*) that is spread by the beetle as it feeds on pine trees. In this study, the mitochondrial genome sequences of four longicorn species (Coleoptera: Cerambycidae: Lamiinae) were determined to further elaborate the phylogenetic relationships of Lamiinae. RT-qPCR was also used to assess the expression of eight mitochondrial protein-coding genes in *M. alternatus* when carrying *B. mucronatus* or not, so as to explore the relationship between these two species. The results showed that expression of mitochondria-encoded genes was elevated in *M. alternatus* beetles that were infected with *B. mucronatus*, suggesting that *B. mucronatus* putatively activates an immune response, which significantly affects the metabolic processes of *M. alternatus*. These results are of significance for further understanding the phylogenetic relationships of longicorn beetles and controlling the spread of pine wilt disease.

**Abstract:**

We determined the mitochondrial gene sequence of *Monochamus alternatus* and three other mitogenomes of Lamiinae (Insect: Coleoptera: Cerambycidae) belonging to three genera (*Aulaconotus*, *Apriona* and *Paraglenea*) to enrich the mitochondrial genome database of Lamiinae and further explore the phylogenetic relationships within the subfamily. Phylogenetic trees of the Lamiinae were built using the Bayesian inference (BI) and maximum likelihood (ML) methods and the monophyly of *Monochamus*, *Anoplophora*, and *Batocera* genera was supported. *Anoplophora chinensis*, *An. glabripennis* and *Aristobia reticulator* were closely related, suggesting they may also be potential vectors for the transmission of the pine wood pathogenic nematode (*Bursaphelenchus xylophilus*) in addition to *M. alternatus*, a well-known vector of pine wilt disease. There is a special symbiotic relationship between *M. alternatus* and *Bursaphelenchus xylophilus*. As the native sympatric sibling species of *B. xylophilus*, *B. mucronatus* also has a specific relationship that is often overlooked. The analysis of mitochondrial gene expression aimed to explore the effect of *B. mucronatus* on the energy metabolism of the respiratory chain of *M. alternatus* adults. Using RT-qPCR, we determined and analyzed the expression of eight mitochondrial protein-coding genes (*COI*, *COII*, *COIII*, *ND1*, *ND4*, *ND5*, *ATP6*, and *Cty b*) between *M. alternatus* infected by *B. mucronatus* and *M. alternatus* without the nematode. Expression of all the eight mitochondrial genes were up-regulated, particularly the *ND4* and *ND5* gene, which were up-regulated by 4–5-fold (*p* < 0.01). Since longicorn beetles have immune responses to nematodes, we believe that their relationship should not be viewed as symbiotic, but classed as parasitic.

## 1. Introduction

Cerambycidae, also known as longicorn beetles, are a very large group within the Coleoptera with over 4000 genera and 35,000 species world-wide and are important components of forest ecosystems [1,2]. Lamiinae is considered to be the most diverse subfamily in Cerambycidae, with about 20,000 described species classified into 80 tribes [3]. The longicorn beetles of Lamiinae are widely distributed and most of the species are major pests in agriculture and forestry. Scholars have formerly relied heavily on morphology [4,5], anatomy and physiology [6], and other methods to establish relationships among species and create classification systems [7]. However, the evolutionary relationships among Lamiinae are hard to describe based on traditional morphological data, but with the development of molecular markers, these relationships can now be assessed with much greater precision [8]. Previous studies have used phylogenetic methods to assess the relationships within the subfamilies of Cerambycidae, and the monophyly of Lamiinae has been corroborated [3,9,10,11]. However, very little other information on mitochondrial genomes has been reported for this subfamily, thus, we aim to enrich the database of Lamiinae and provide useful clues for the internal phylogenetic relationship of Lamiinae. 

*Monochamus alternatus*, belongs to the subfamily Lamiinae. In East Asia, *M. alternatus* is believed to be the main vector for the pathogenic nematode *Bursaphelenchus xylophilus* [12] that infects pine trees and causes pine wilt disease [13]. *B. xylophilus* is transported to host pine trees by *Monochamus* beetles and after nematodes develop rapidly and form dispersal juveniles, these juveniles enter the tracheal system of the beetle for transport to new pine trees hosts, thus spreading the disease [14]. *Bursaphelenchus mucronatus*, a sister species to *B. xylophilus*, is a migratory endoparasitic nematode [14,15,16]. However, as a native sympatric sibling nematode to *B. xylophilus*, in both its morphological and biological characteristics [17], *B. mucronatus* shows weaker pathogenicity to forests [18]. However, various scholars believe that *B. mucronatus* can also cause pine wilt disease, and that this species jointly forms a complex with *B. xylophilus* [19,20]. Previous studies have revealed the physiological changes and symptom development of pine wilt disease, such as decreased photosynthesis, cambium destruction, traumatic resin canal formation, etc. [21]. Much attention has been given to the effects on the health of pine trees, but few studies have focused on the relationships between longicorn beetles and nematodes.

Mitochondria are mainly known for their crucial roles in aerobic ATP production involving the citric acid cycle and oxidative phosphorylation [22]. However, these organelles are involved in a variety of other processes including apoptosis and immunity [23]. In addition, the mitochondrial genome is widely considered to be a highly informative molecular marker for comparative genomic research [24,25], and is widely used to study phylogenetic relationships due to its maternal inheritance and high evolutionary rate [26]. There have already been several studies related to mitochondrial gene expression [27,28,29,30]. Previous studies have already demonstrated that gene expression in *M. alternatus* was different when infected with *B. xylophilus* [31,32]. Zhou et al. [31] proved that *M. alternatus* infected with *B. xylophilus* showed an increase in the expression of antioxidant genes in order to obtain immune tolerance. In addition, Ning et al. [32] found that there were many differentially expressed microRNAs (miRNAs) in *M. alternatus* with nematode infections and the expression of neuron-related miRNAs increased significantly. However, current research on mitochondrial genes related to *M. alternatus* and pine wood nematodes has focused only on genome sequencing and the regulation of nuclear genes, and there has not been much work on mitochondrial gene expression. Li et al. [33] indicated that there was no difference in the transcript level of *M. alternatus* infected with *B. xylophilus* or not. Considering that mitochondria are essential for the production of adenosine triphosphate (ATP) through oxidative phosphorylation [34], the energy metabolism of the longicorn beetles may change and manifest as a change in the transcript level of mitochondrial protein coding genes when carrying nematode. In the present study, the mitochondrial genome was not only used to study the phylogenetic relationships of Lamiinae, but also to figure out the relative expression of mitochondrial protein coding genes in response to *B. mucronatus*.

The present study aims to determine if there is a difference in mitochondrial gene expression in *M. alternatus* when infected with *B. mucronatus*. We examine how *B. mucronatus* changes the energy metabolism of *M. alternatus*, and further explore the parasitic relationship. We also provide new ideas about pine wilt disease by studying mitochondrial gene expression responses when *M. alternatus* beetles are infected by pine wood nematodes. Because molecular data on the species within the subfamily Lamiinae is still scarce, we also sequenced four mitochondrial genomes from Lamiinae species in order to enrich the mitochondrial genome sequence database and we built phylogenetic trees to explore the phylogenetic relationships within the Lamiinae subfamily.

## 2. Materials and Methods

### 2.1. Sampling Collection and Microscopic Examination

Samples of live *M. alternatus* were captured by traps set in the Taihuyuan pine wood nematode epidemic area of Lin’an (30°35′ N, 119°58′ E), Zhejiang Province, China. Samples of *Aulaconotus atronotatus* were captured from Jinxiu, Guangxi Province (24°11′ N, 109°58′ E), and *Apriona germari* along with *Paraglenea fortunei* were captured from Zhejiang Normal University, Jinhua (29°14′ N, 119°64′ E), Zhejiang Province, China. After morphological identification, the specimens of *M. alternatus* were then cultivated in the laboratory under artificial feeding conditions. Cultivation was in a plastic incubator that was placed in an artificial climate box; fresh Masson pine was provided every 3 days, the temperature in the box was maintained at 20–25 °C and relative humidity was 60–70%. Specimens of the other three longicorn beetles were deposited at −40 °C in the Animal Specimen Museum, College of Life Sciences and Chemistry, Zhejiang Normal University, China. After experimentation, all the pine wood was incinerated.

All *M. alternatus* were quickly dissected after a week and small pieces of abdominal tissue, thorax flight muscle, and intestinal tract were taken for microscopical examination. We used an OLYMPUS-SZX16 stereo microscope to observe abdominal tissue samples to directly determine the presence/absence of nematodes. If nematodes were found in *M. alternatus* tissue, then tissues from that individual were assigned to the experimental group whereas beetles without nematodes were placed into the control group. The excised digestive tract and flight muscle tissue samples were quickly frozen in liquid nitrogen and stored in an ultra-low temperature refrigerator at −80 °C until use.

### 2.2. DNA Extraction, Sequencing, and Molecular Identification

Total genomic DNA was extracted from thorax flight muscle tissues of each specimen by an Ezup Column Animal Genomic DNA purification Kit (Sangon Biotech Company, Shanghai, China). After bright and clear bands were detected with 1% agarose gel electrophoresis, the DNA was then stored at −20 °C. Cycling conditions and reaction volume of PCR amplifications were as in Cheng et al. and Gao et al. [35,36]. All PCR products were obtained using an automated DNA sequencer (ABI 3730) from Sangon Biotech Company (Shanghai, China) for both mitochondrial strands.

Referring to the method of amplifying insect genomes by Simon et al. [37] and combined with the *M. alternatus* mitochondrial genome published in the GenBank database, Clustal W software in Mega 7.0 [38] was used to align the primer sequences. Eleven pairs of universal primers were modified and 11 adjacent or overlapping fragments were amplified [38,39,40,41]. Using qualified DNA samples as templates, the mitochondrial genes of *M. alternatus* obtained from the Lin’an District of Hangzhou were sequenced via PCR. Primers were also used to amplify the mitochondrial genome fragments of the nematodes collected and were then further modified to distinguish between *B. xylophilus* and *B. mucronatus* [42]. After using these detection primers in PCR experiments, the appearance of a bright and clear band after electrophoresis meant that sample contained the corresponding nematode species. This step also eliminated the possibility that the control samples were carrying nematodes. Through this step, we ensured that the longicorn beetles in the control group do not contain nematodes, and that the beetles in the experimental group can be detected as *B. mucronatus* by both microscope examination and PCR.

### 2.3. Sequence Annotation and Analyses

All mitochondrial genome sequences were manually proofread and spliced using DNASTAR Package v.6.0 software [43] and tRNA gene annotation was completed through the online website MITOS (http://mitos.bioinf.uni-leipzig.de/index.py, accessed on 15 January 2021) [44]. The complete sequences of some Cerambycidae species were obtained from the GenBank database [1,2,11,45,46,47,48,49,50,51,52,53], and homologous sequence alignments were performed by the Clustal W program of Mega 7.0 [38] to determine the positions of the two rRNA genes and the AT-rich region. The four mitogenomes were deposited in GenBank with the accession numbers MW858148, MW858150-MW858152. Also, the nucleic acid sequences of the 13 protein-coding genes (PCGs) were translated into amino acid sequences on the basis of the invertebrate genetic codes [54]. Mega 7.0 [38] software was used to find the open reading frame of each of the 13 PCGs, and to calculate the AT content and codon usage rate of the newly determined mitochondrial sequences. The formula used for skew analysis of gene composition was as follow: AT-skew = (A − T)/(A + T) and GC-skew = (G − C)/(G + C) [55]. The relative synonymous codon usage (RSCU) of four longicorn beetle mitochondrial genomes was calculated using PhyloSuite [56].

### 2.4. Comparison of Protein Coding Genes and Phylogenetic Analyses

To study the phylogenetic relationships of Cerambycidae, a nucleotide dataset (13P33) of the 13 protein-coding genes of 33 mitogenomes was used according to Zhang et al. [57] (Table 1). Newly sequenced mitogenomes of *Au. atronotatus*, *Ap. germari*, *P. fortunei* and *M. alternatus* were included, with *Sympiezomias velatus* and *Naupactus xanthographus* selected as outgroups. The Clustal W program of Mega 7.0 software was used to align the amino acid sequences of the protein-coding genes [38], the conserved regions were selected by Gblock 0.91b [58], and PartionFinder 1.1.1 [59] was used to select partitions based on Bayesian information criterion (BIC). The phylogenetic relationships were analyzed by the Bayesian inference (BI) and maximum likelihood (ML) methods. The partition schemes and best-fit models selected for each data set are provided in Table 2. We used a GTR + I + G model to do ML and BI analyses. ML analysis was performed by RAxML 8.2.0 software based on the GTRGAMMAI best model, and branch confidence was obtained by rapid inference using 1000 repetitions [60]. The BI analysis was run by MrBayes 3.2 [61] based on the GTR + I + G model through 10 million generations, with the first 25% of generations removed as burn in, and the mean standard deviation of Bayesian split frequency was less than 0.01.

### 2.5. RNA Isolation and cDNA Synthesis

Samples (50–100 mg) of *M. alternatus* intestines frozen at ultra-low temperature were used for RNA extraction according to the instructions of the TaKaRa MiniBEST Universal RNA Extraction Kit (Takara, Japan). The integrity of RNA was verified by the presence of sharp bands for *28S* and *18S rRNA* using a 1% agarose gel stained with Goldview and run at 130 V, 120 mA for 25 min [62]. Then, the samples were frozen at −80 °C until use. We performed reverse transcription according to the PrimeScript™ RT Master Mix kit instructions (Takara, Japan). Reverse transcription reaction conditions were: 37 °C, reverse transcription for 15 min; 85 °C, reverse transcriptase inactivation for 5 s.

### 2.6. RT-qPCR

We used DNASTAR [43] and Primer Premier 5 software [63] to design primers according to the *M. alternatus* sequence downloaded from the NCBI website and the newly obtained sequence of *M. alternatus* in Lin’an, Hangzhou. The *β-actin* gene was selected as the reference gene [62,64] to design the internal reference primer of *M. alternatus*. The amplicon was synthesized by Sangon Biotech Company (Shanghai, China), followed by screening of the quantitative primers for 13 protein-coding genes. Due to the strong AT skew and the length of gene fragments in 5 protein-coding genes, their primers failed to amplify. However, the remaining eight protein-coding genes could be used for RT-qPCR experiments (Table 3). The primers shown in Table 3 were screened by RT-qPCR reactions, and then gene expression in the intestine of *M. alternatus* was measured in both experimental and control groups. The genes corresponding to each primer pair were assessed in three technical replicates. Reactions were carried out in Primer Script TM RT Master Mix (TaKaRa, Tokyo, Japan), with conditions as follows: initial denaturation at 95 °C for 30 s, and 40 cycles (95 °C for 5 s, 60 °C for 30 s) to generate a melt curve.

The relative expression of mRNA in the intestinal tract of *M. alternatus* at a specific stage was analyzed by the 2^−ΔΔCt^ method and normalized to the *β-actin* gene. We used SPSS 21.0 software to analyze all data, and data was expressed as mean ± standard deviation (mean ± SE). During the data analysis process, the average value of the control group was set to 1. Gene expression of the experimental and control groups was pre-compared using the *t*-TEST function, with *p* < 0.05 considered statistically significant. Finally, Origin 8.0 software was used to plot results.

## 3. Results

### 3.1. Nematode Identification

After the microscopic examination, we found that some abdominal tissues contained nematodes, so we proceeded to molecular identification. We used specific primers for detection of *B. xylophilus* and *B. mucronatus* genes and performed PCR analysis on the DNA template of *M. alternatus*. The results showed that only the *B. mucronatus* primers produced bright and clear bands after gel electrophoresis. Therefore, it was determined that the *M. alternatus* specimens that were collected contained only *B. mucronatus* nematodes. *M. alternatus* tissue samples from individuals without nematodes were separated into the control group, and those containing *B. mucronatus* were placed in the experimental group.

### 3.2. Components of the Mitochondrial Genomes

We obtained one complete and three almost complete mitogenomes of Lamiinae species representing four genera (*Aulaconotus*, *Apriona*, *Paraglenea*, and *Monochamus*) (Supplementary Materials, Tables S1–S4). The complete mitochondrial genome of *P. fortunei* was 15,496 bp in length, and the mitogenomes of *Au. atronotatus*, *Ap. germari*, and *M. alternatus* were 14,491 bp, 14,858 bp, and 14,189 bp in length, respectively.

**Figure 1:**
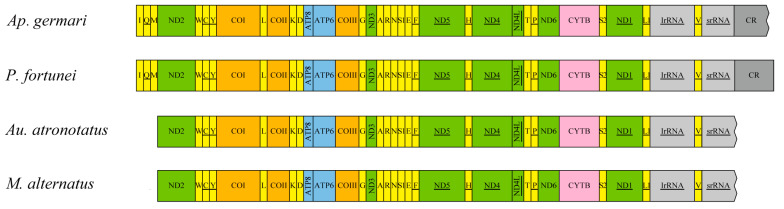
Complete mitochondrial genome structures of *Au. atronotatus*, *Ap. germari*, *P. fortune* and *M. alternatus*. Genes encoded by the L-strand are underlined whereas those without underline are encoded on the H-strand. Different colored boxes represent different genes.

The mitogenome of *P. fortunei* contained 13 protein-coding genes, 22 tRNAs, two rRNAs and an A + T rich region, the same as the ancestral mitochondrial genome (Figure 1). The A + T content, AT-skew and GC-skew of corresponding regions (mitogenome, PCGs and rRNAs) of each longicorn beetle species were calculated and are shown in Table 4. The nucleotide composition of the four longicorn beetles mitogenomes was strongly biased towards A and T, which made up 74.2% (*Au. atronotatus*) to 78.4% (*M. alternatus*) of the base pairs. All tRNAs of these longicorn beetles showed a typical cloverleaf secondary structure except for *trnS1* (AGA) in *Au. atronotatus* and *Ap. Germari*, which formed a simple loop instead of the dihydrouridine (DHU) arm (Figures S1–S4).

**Figure 2:**
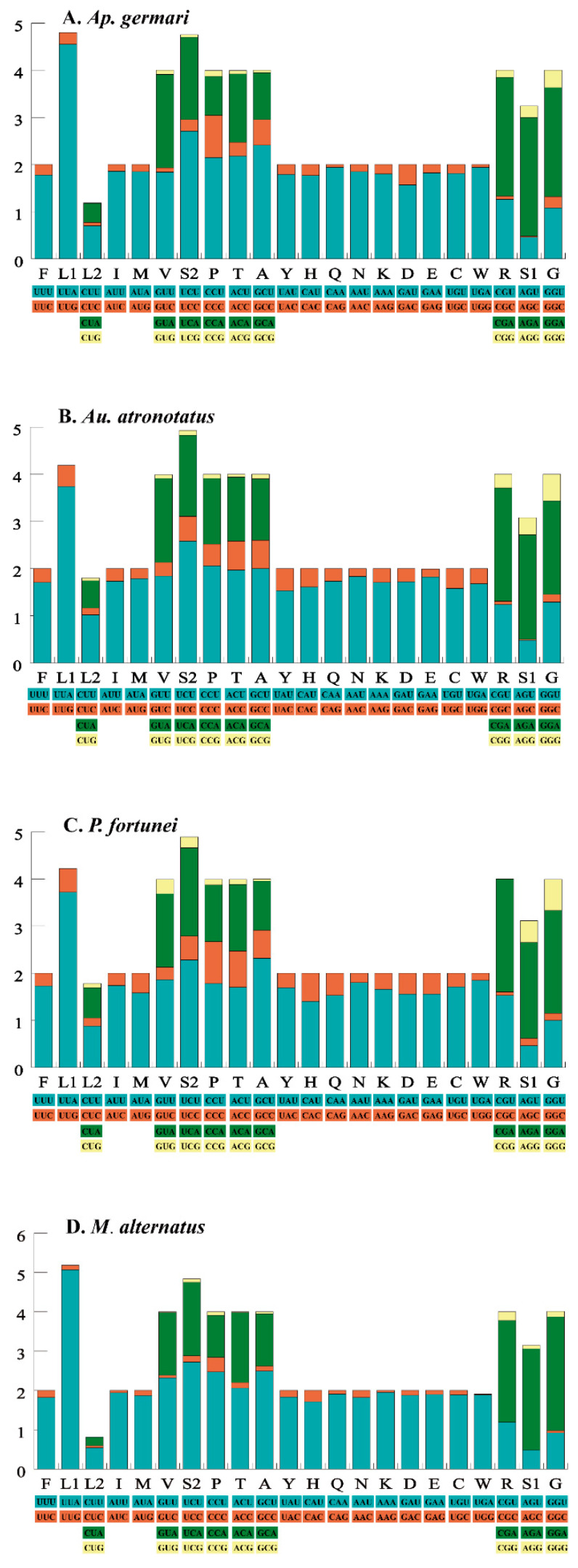
The relative synonymous codon usage (RSCU) of the 13 protein-coding genes. Codon families are provided on the x-axis along with the different combinations of synonymous codons that code for that amino acid. RSCU is defined on the Y axis.

Among the four sequenced mitochondrial genomes, 11 protein-coding genes used the typical invertebrate start codon ATN, whereas TTG and GTA were used as the initiation codon in *ND1* and *ND3*. Typical stop codons such as TAA and TAG were widely used. However, we also observed an incomplete stop codon T in five genes: *COI* (*Au. atronotatus* and *M. alternatus*), *COII* (*Au. atronotatus*, *Ap. germari*, *P. fortunei* and *M. alternatus*), *COIII* (*Au. atronotatus* and *M. alternatus*), *ND4* (*Ap. germari*, *P. fortunei* and *M. alternatus*) and *ND5* (*Au. atronotatus*, *Ap. germari*, *P. fortunei* and *M. alternatus*). The RSCU of each of the mitogenomes was calculated and the results indicated that the main codons used were highly similar in the four longicorn beetles (Figure 2). The most used codons (>284) in the PCGs of these mitogenomes were UUA (Leu), AUU (Ile) and UUU (Phe).

### 3.3. Phylogenetic Analyses

**Figure 3:**
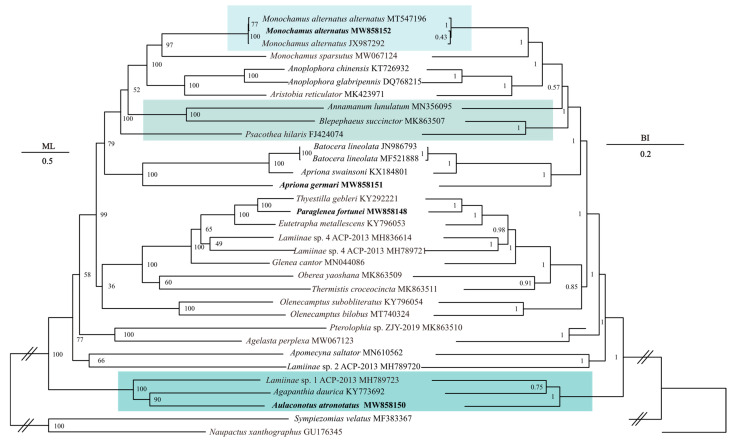
Phylogenetic tree of the relationships among 33 species of longicorn beetles based on the nucleotide dataset of the 13 mitochondrial PCGs. *S. velatus* and *N. xanthographus* was used as the outgroup. The numbers above branches specify posterior probabilities as determined from BI (left) and bootstrap percentages from ML (right). The GenBank accession numbers of all species are shown in the figure. The species in bold are sequenced in this study, and the species with colored frame are the species with different topology between ML and BI trees.

Based on the 13 mitochondrial protein-coding genes, the phylogenetic relationships within the subfamily Lamiinae were inferred from BI and ML analyses (Figure 3). The results showed that both ML and BI trees within Lamiinae split into two branches: (1) the clade consisting of *Lamiinae* sp. 1 ACP-2013, *Agapanthia daurica* and *Au. atronotatus* was the earliest diverging clade, and (2) the other 28 species gathered together in another branch. Lamiinae and the outgroup were well separated. In addition, the monophyly of *Monochamus*, *Anoplophora*, *Batocera* genera was supported.

The topological structure of the ML and BI trees was basically the same but with a few differences (Figure 3). In the ML tree, the clade of (*M. alternatus* MT547196 + *M. alternatus* MW858152) was supported, whereas in the BI tree a clade of (*M. alternatus* JX987292 *+ M. alternatus* MW858152) was supported. ML analysis showed the clade of (*Lamiinae* sp. 1 ACP-2013 + (*Ag. daurica* + *Au. atronotatus*)) was the most basal clade within the Lamiinae subfamily, and in the BI tree the relationship of basal clade was shown as ((*Lamiinae* sp. 1 ACP-2013 + *Ag. daurica*) + *Au. atronotatus*). Moreover, *Psacothea hilaris* was the sister clade of ((*Annamanum lunulatum* + *Blepephaeus succinctor*) + ((*Aristobia reticulator* + *Anoplophora* spp.) + *Monochamus* spp.)) in ML analysis, but in BI analysis, we found that the clade of (*An. lunulatum* + *Bl. succinctor*) was the sister clade of (*Psacothea hilaris* + ((*Aristobia reticulator* + *Anoplophora* spp.) + *Monochamus* spp.)).

### 3.4. Transcriptions of Mitochondrial Protein-Coding Genes

**Figure 4:**
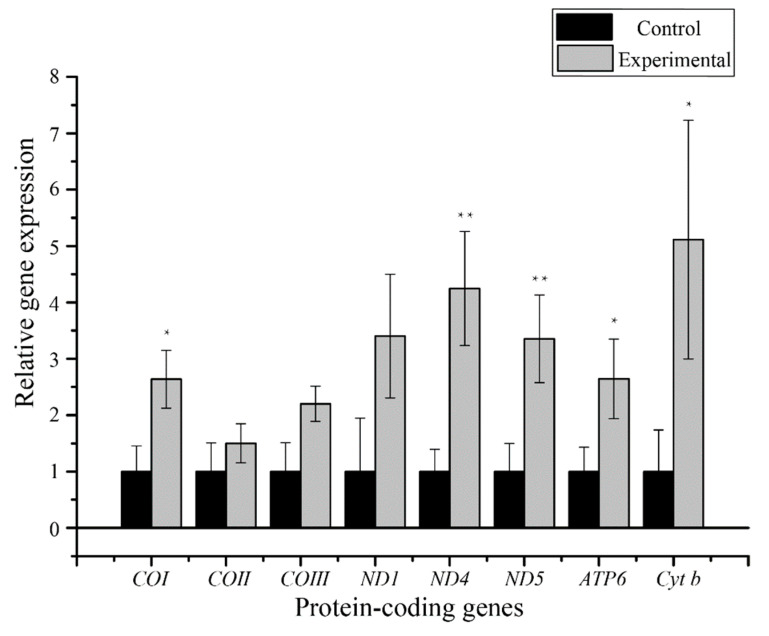
The expression of eight protein-coding genes from *M. alternatus* carrying *B. mucronatus* or not. The x-axis shows gene names, and the y-axis shows relative gene expression. Black columns show controls, standardized to 1.0; gray columns show the corresponding experimental group (carrying *B. mucronatus*). Asterisks indicate significantly different expression: *, *p* < 0.05); **, *p* < 0.01.

The relative transcription of eight mitochondrial protein-coding genes from the intestines of control and experimental groups of *M. alternatus* were quantitatively determined by RT-qPCR to assess the effects of the presence versus absence of *B. mucronatus* nematodes on the transcription of the beetle genes (Figure 4). The results indicated that in the presence of *B. mucronatus*, transcript levels of *COI*, *COII*, *COIII*, *ND1*, *ND4*, *ND5*, *ATP6* and *Cyt b* were up-regulated in *M. alternatus* to varying degrees. The transcription level of *ND4* and *ND5* genes increased by 4.25 ± 1.01, and 3.35 ± 0.78-fold, respectively (*p* < 0.01). Transcript levels of *Cyt b*, *COI*, and *ATP6* genes in beetle intestine also increased significantly by 5.11 ± 2.12, 2.64 ± 0.51 and 2.64 ± 0.71-fold, as compared to the control group, respectively (*p* < 0.05). The remaining three genes (*COII*, *COIII*, *ND1*) showed no significant differences in their transcription levels between control and nematode-infected groups.

## 4. Discussion

### 4.1. Phylogenetic Analyses

The Lamiinae subfamily is widely distributed and contains the largest number of species among the Cerambycidae, but the phylogenetic relationships within Lamiinae are still controversial [2]. However, the subfamily is considered to be a monophyletic group, as has been shown in previous studies [2,9,10,11]. In our study, two species within the subfamily Entiminae were selected as the outgroups, and the results showed that all the Lamiinae species were well separated with a high degree of confidence. Both ML and BI phylogenetic trees showed the formation of two large branches within the Lamiinae subfamily (Figure 3). *Batocera lineolata* (JN986793 and MF521888) was closely related to *Apriona swainsoni*, which was consistent with the results of Wang et al. [2]. We also found that the clade of ((*An. chinensis* + *An. glabripennis*) + *Aristobia reticulator*) was a sister clade to the genus *Monochamus* with 100% confidence both in ML and BI analyses. These longicorn beetles showed a close phylogenetic relationship, suggesting that *An. chinensis*, *An. glabripennis* and *Ar. reticulator* may all be potential vectors for the transmission of pine wood nematodes. Our study increases the richness of the Cerambycidae genome information and can assist in phylogenetic, molecular systematics and evolutionary studies of Cerambycidae. However, molecular data on species of Lamiinae are still scarce and more information is needed to fully explore the phylogeny within Lamiinae.

### 4.2. Mitochondrial Gene Expression

The relative transcription of eight mitochondrial protein-coding genes in the intestines of the control and experimental groups of *M. alternatus* was quantitatively determined by RT-qPCR to compare the effects of the presence versus absence of *B. mucronatus* nematodes on mitochondrial gene expression in *M. alternatus*. The results showed that transcription of all eight genes in the experimental group was up-regulated to varying degrees.

Mitochondria are essential to the process of generating adenosine triphosphate (ATP) through oxidative phosphorylation [34]. The mitochondrial respiratory chain responsible for generation of cellular ATP consists of five multi-subunit enzyme complexes (Complex I–V) located at the inner mitochondrial membrane. Four of these, Complexes I, III, IV and V, are bipartite and consist of subunits derived from both mitochondrial DNA (mtDNA) and nuclear DNA [65,66,67]. Thirteen protein-coding genes are encoded by the mitogenome and whether they are correctly expressed is very important for maintaining mitochondrial respiratory chain function [68]. Changes in the levels of mtDNA-encoded mRNA occur rapidly in response to changes in energy requirements of the cell [66]. When infected with *B. mucronatus*, the expression of *COI*, *ND4*, *ND5*, *ATP6*, and *Cyt b* genes in *M. alternatus* were all significantly up-regulated, and *ND4* and *ND5* genes increased by 4.25 ± 1.01 and 3.35 ± 0.78-fold compared to control groups (*p* < 0.01). These results support the viewpoint proposed by Li et al. [33] that a large number of stress-responsive genes were up-regulated in *M. alternatus* when infected with pine wood nematodes. These five genes are important subunits of Complex I, III, IV, and V and can significantly affect metabolic energy processes. *ND4* and *ND5* genes are believed to have originated from a common ancestor and have similar functions in the synthesis of Complex I [69]. Complex I is the main point for electrons to enter the electron transport chain, and is key for cells to produce large amounts of ATP through oxidative phosphorylation [34]. Complex I is also one of the main sources of reactive oxygen species (ROS) [67,70]. The *Cyt b* gene is responsible for electron transfer in Complex III. Cytochrome bc1 encoded by *Cyt b* is the only subunit of Complex III that is encoded by a mitochondrial gene, and has an important relationship with the composition of the functional Complex III [71]. The *COI* gene is one of the subunits of Complex IV [72]. Complex IV catalyzes the oxidation of cytochrome C and the reduction of oxygen to water, it is an enzyme at the end of the electron transport chain and it also functions as a proton pump [73]. The *ATP6* gene is one of the subunits of Complex V. Under normal physiological conditions, this multi-subunit protein utilizes energy in the form of the proton gradient, generated by the electron transport chain, to phosphorylate ADP to form ATP [74].

*B. mucronatus* and *B. xylophilus* belong to the same genus, and in the past, *B. mucronatus* has attracted little attention as compared with *B. xylophilus*. Some previous studies have already shown that the expression of several genes in *M. alternatus* was altered when infected by *B. xylophilus*. For example, Zhou et al. [31] showed that in order to obtain immune tolerance, *M. alternatus* infected with *B. xylophilus* would induce an increase in the expression of antioxidant genes. Through the increased expression of these antioxidant genes, the level of ROS in the trachea of longicorn beetles increases and reaches a state of equilibrium [31]. Coincidentally, as one of the main sources of ROS, the genes *ND4* and *ND5*, which code for two subunits of Complex I, were found to be significantly up-regulated in this study. Besides, mitochondria can simultaneously produce ATP and macromolecule synthesis, which enables mitochondria to meet the metabolic needs of different immune cells [75]. Accumulating evidence has shown that cellular energy metabolism pathways are altered during the differentiation and activation of immune cells, revealing mechanisms of metabolic regulation and roles in immune functions [76]. In our study, analysis of the expression of mitochondrial-encoded genes of the electron transport chain complex showed up-regulation of transcript levels in *M. alternatus* infected with *B. mucronatus*, indicating increased synthesis of protein subunits in Complexes I, III, IV, and V. This may indicate that *M. alternatus* is gaining immune tolerance to the parasitic nematode. Besides, a hypothesis has been proposed that metabolism can be an input to the system and act as a signal to control the immune functions of a cell [77]. Loftus et al. also proposed that cellular metabolism has direct roles in regulating immune cell function. Ning et al. showed that *M. alternatus* with and without *B. xylophilus* nematodes had different metabolic characteristics [32]. There were many differentially expressed microRNAs in *M. alternatus* infected with *B. xylophilus* and their functions were mostly directed towards regulating genes associated with metabolism and immunity [32], which is also consistent with the significant increase in mitochondrial gene expression described in our research results. The expression of certain miRNAs related to apoptosis, material metabolism, and olfactory neurons in *M. alternatus* with *B. xylophilus* increased significantly [32]. Li et al. found that in *B. xylophilus* infected beetles the differentially expressed gene functions in adult *M. alternatus* tissues mainly point to pressure regulation in the trachea, hormone response, and tissue and DNA repair [33]. By enhancing the expression of various genes, the tolerance of *M. alternatus* to *B. xylophilus* is improved, and a stable state of life is also maintained [22].

Combining these research results with ours, indicated that the up-regulated mitochondrial protein coding genes expressed in long beetles carrying *B. mucronatus* may play a key role in the process of carrying nematodes. The relationship formed between *M. alternatus* and *B. mucronatus* could be related to the expression of mitochondrial protein-coding genes. The results showed that when carrying nematodes, there was an enhancement in mitochondrial metabolism, and the up-regulated genes were all related to immune function, suggesting that the immune function of longicorn beetles is affected and they could have created immune measures against nematodes to protect themselves. Previous studies have shown that in the process of *M. alternatus* responding to pine wood nematodes, *M. alternatus* is in a state of suppressed immunity as a whole. Some immune genes in the epidermis and trachea are up-regulated, and the expression level of most immune genes remains unchanged. *M. alternatus* maintains immune homeostasis between the two through the regulation of ROS [31,78]. Some researchers believe that longicorn beetles and nematodes have a symbiotic relationship [33,79]. However, as the existence of *B. mucronatus* increased the transcription level of immune-related genes in *M. alternatus*, which manifested as those *M. alternatus* maintaining a high metabolic level in the intestine, we prefer to suggest the relationship between them is more like a parasite. We speculate that more efforts were made by *M. alternatus* to offset the negative effects of nematodes. Werner also suggested through molecular phylogeny that the relationship between nematodes and beetles should be host switching rather than nematode-beetle coevolution [80]. The other mechanisms need to be studied in the future.

## 5. Conclusions

The complete mitochondrial genome sequence of *P.* *fortunei* and three nearly complete mitogenomes of *Au.* *atronotatus*, *M.* *alternatus*, *Ap. germari* were determined. The phylogenetic tree supported the monophyly of *Monochamus*, *Anoplophora*, *Batocera* genera. Since the clade consisting of *An. chinensis*, *An. glabripennis* and *Ar. reticulator* was a sister clade to the one containing *Monochamus* with 100% confidence, it is suggested that all four of these species may be potential vectors for the transmission of pine wood nematodes. All of the eight mitochondrial genes that were assessed showed an up-regulation trend, with *CO I*, *ATP6* and *Cyt b* being significantly up-regulated (*p* < 0.05) with transcript levels increased by 5.11 ± 2.12, 2.64 ± 0.51 and 2.64 ± 0.71-fold, respectively. *ND4* and *ND5* genes were also significantly up-regulated (*p* < 0.01) with 4.25 ± 1.01 and 3.35 ± 0.78fold increases, respectively. Since five genes corresponding to important subunits of Complex I, III, IV, and V were affected, it can be proposed that the increase in gene expression is a response to the changing demands of cellular energy requirements. To sum up, the *M. alternatus* that carries the *B. mucronatus* nematode still maintains a high metabolic level in the intestine, and this could be related to an autoimmune or symbiotic relationship of longicorn beetles against nematodes or it may indicate a parasitic relationship between longicorn beetles and nematodes.

## Figures and Tables

**Table 1:** Species sample information used in the phylogenetic tree construction in this study.

Subamily	Genus	Species	GenBank No.	References
Lamiinae	*Monochamus*	*Monochamus alternatus alternatus*	MT547196	Liao (2020)
		*Monochamus alternatus*	MW858152	**This study**
		*Monochamus alternatus*	JX987292	Wang (2012)
		*Monochamus sparsutus*	MW067124	Wu (2020)
	*Anoplophora*	*Anoplophora chinensis*	KT726932	Li (2015)
		*Anoplophora glabripennis*	DQ768215	An (2006)
	*Aristobia*	*Aristobia reticulator*	MK423971	Behere (2019)
	*Psacothea*	*Psacothea hilaris*	FJ424074	Kim (2009)
	*Annamanum*	*Annamanum lunulatum*	MN356095	Dai (2020)
	*Blepephaeus*	*Blepephaeus succinctor*	MK863507	Wang (2019)
	*Batocera*	*Batocera lineolata*	JN986793	Wang (2011)
		*Batocera lineolata*	MF521888	Liu (2017)
	*Apriona*	*Apriona swainsoni*	KX184801	Que (2016)
		*Apriona germari*	MW858151	**This study**
	*Thyestilla*	*Thyestilla gebleri*	KY292221	Yang (2016)
	*Paraglenea*	*Paraglenea fortunei*	MW858148	**This study**
	*Eutetrapha*	*Eutetrapha metallescens*	KY796053	Yang (2017)
		*Lamiinae* sp. *4 ACP-2013*	MH836614	Crampton-Platt (2015)
		*Lamiinae* sp. *4 ACP-2013*	MH789721	Crampton-Platt (2015)
	*Glenea*	*Glenea cantor*	MN044086	Wang (2019)
	*Oberea*	*Oberea yaoshana*	MK863509	Wang (2019)
	*Thermisti*	*Thermistis croceocincta*	MK863511	Wang (2019)
	*Olenecamptus*	*Olenecamptus subobliteratus*	KY796054	Yang (2017)
		*Olenecamptus bilobus strain fentianniu*	MT740324	Dong (2020)
	*Pterolophia*	*Pterolophia* sp. *ZJY-2019*	MK863510	Wang (2019)
	*Agelasta*	*Agelasta perplexa*	MW067123	Chen (2020)
	*Apomecyna*	*Apomecyna saltator*	MN610562	Nie (2021)
		*Lamiinae* sp. *2 ACP-2013*	MH789720	Crampton-Platt (2015)
		*Lamiinae* sp. *1 ACP-2013*	MH789723	Crampton-Platt (2015)
	*Agapanthia*	*Agapanthia daurica*	KY773692	Yang (2017)
	*Aulaconotus*	*Aulaconotus atronotatus*	MW858150	**This study**
	*naupactus*	*Naupactus xanthographus*	GU176345	Song (2010)
	*sympiezomias*	*Sympiezomias velatus*	MF383367	Tang (2017)

**Table 2:** The partition schemes and best-fitting models selected.

Nucleotide Sequence Alignments		
Subset	Subset Partitions	Best Model
Partition 1	*COX1*_codon1, *COX2*_codon1, *ATP6*_codon1, *COX3*_codon1, *CYTB*_codon1	GTR + I + G
Partition 2	*COX1*_codon2, *COX3*_codon2, *ATP6*_codon2, *COX2*_codon2, *CYTB*_codon2	TVM + I + G
Partition 3	*COX1*_codon3, *COX2*_codon3, *ATP6*_codon3, *ATP8*_codon3	TIM + G
Partition 4	*ATP8*_codon2, *ND2*_codon1, *ND3*_codon1, *ND6*_codon1, *ATP8*_codon1	GTR + I + G
Partition 5	*ND6*_codon3, *COX3*_codon3, *ND3*_codon3, *CYTB*_codon3	HKY + G
Partition 6	*ND4L*_codon1, *ND1*_codon1, *ND5*_codon1, *ND4*_codon1	TVM + I + G
Partition 7	*ND4L*_codon2, *ND1*_codon2, *ND5*_codon2, *ND4*_codon2	GTR + I + G
Partition 8	*ND1*_codon3	HKY + G
Partition 9	*ND6*_codon2, *ND2*_codon2, *ND3*_codon2	TVM + I + G
Partition 10	*ND2*_codon3	TRN + G
Partition 11	*ND5*_codon3, *ND4*_codon3, *ND4L*_codon3	HKY + G

**Table 3:** Primer sequences of eight mitochondrial protein-coding genes and *β-actin* gene designed for RT-qPCR experiment in this study.

Gene	Forward Primer	Forward Primer
*β-actin*	CTCAACCCCAAGGCTAACC	CACCATCTCCAGAGTCCAAT
*COI*	CTC(T)TTACCTCCTTCTTTAACTC	CAACTGATGAACCTCTATGAG
*COII*	GATGCAACTCCTGGACGAT	ATCTATGATTGGCACCACAA
*COIII*	AGAGCCTTATCTCCTAGAATTG	GCTCAAGTTACTGTTAATCCTG
*ND1*	ATTATC(T)GCAAATCCACCTCT	TAGCAGAAACTAATCGTACTCC
*ND4*	GAAGGAGGAGCAGCCATA	CTTCAGGTTTATTTTGTTTAGC
*ND5*	TAGTAAAGCAACATCCCCA	TATTAGGGTGAGATGGTTTAG
*ATP6*	TTAGTACCTCAAGGAACTCC	GATAATCGAACTGCCAATGT
*Cyt b*	ATCATTCTGAGGAGCAACTG	TGAAAGGTAAAAAATCGTGT

**Table 4:** Base composition of four Lamiinae mitochondrial genomes.

Species	A + T (%)				AT-Skew				GC-Skew			
	Mito	PCGs	rRNAs	tRNAs	Mito	PCGs	rRNAs	tRNAs	Mito	PCGs	rRNAs	tRNAs
*A. atronotatus*	74.2	73.5	76.8	78.5	0.019	−0.15	−0.052	0.029	−0.0222	−0.034	0.401	0.133
*A. germari*	76.8	76.3	77.7	77.2	0.013	−0.151	−0.028	0.036	−0.0208	−0.002	0.409	0.154
*P. fortunei*	74.3	73	75.7	76	0.038	−0.152	−0.062	0.031	−0.287	−0.014	0.472	0.116
*M. alternatus*	78.4	77.7	82.1	79.9	0.006	−0.152	−0.023	0.034	−0.153	0.026	0.342	0.112

## Data Availability

Not applicable.

## Supplementary Materials

The following are available online at https://www.mdpi.com/article/10.3390/insects12050453/s1, Table S1. Location of features in the mitochondrial genome of *Au. atronotatus*. Table S2. Location of features in the mitochondrial genome of *Ap. germari*. Table S3. Location of features in the mitochondrial genome of *P. fortune*. Table S4. Location of features in the mitochondrial genome of *M. alternatus*. Figure S1. Inferred secondary structures of the tRNA genes in *Au. atronotatus*. Figure S2. Inferred secondary structures of the tRNA genes in *Ap. germari*. Figure S3. Inferred secondary structures of the tRNA genes in *P. fortune*. Figure S4. Inferred secondary structures of the tRNA genes in *M. alternatus*.
