# Supplementary material for: Mitogenome Analysis of Four Lamiinae Species (Coleoptera: Cerambycidae) and Gene Expression Responses by Monochamus alternatus When Infected with the Parasitic Nematode, Bursaphelenchus mucronatus
Source: Insects. 2021 May 14;12(5):453. doi: 10.3390/insects12050453 (PMC8157225; doi:10.3390/insects12050453)
Supplement: Supplementary file 1 [file insects-12-00453-s001.zip › insects-1211092-supplementary/Supplementary Materials/Table S1 .docx]

| Gene | Strand | Position | Length  (nuc.) | Anticodon | Start  codon | Stop  codon | Intergenic  nucleotides |
| --- | --- | --- | --- | --- | --- | --- | --- |
| *ND2* | + | 13-1014 | 1002 |  | ATA | TAA | -2 |
| *tRNA^Trp^* | + | 1013-1078 | 66 | TGA |  |  | -8 |
| *tRNA^Cys^* | - | 1071-1133 | 63 | TGC |  |  | +1 |
| *tRNA^Tyr^* | - | 1135-1199 | 65 | TAC |  |  | -8 |
| *COⅠ* | + | 1192-2734 | 1543 |  | ATT | T | 0 |
| *tRNA^Leu2^* | + | 2735-2799 | 65 | TTA |  |  | 0 |
| *COⅡ* | + | 2800-3484 | 685 |  | ATT | T | +1 |
| *tRNA^Lys^* | + | 3486-3555 | 70 | AAA |  |  | -2 |
| *tRNA^Asp^* | + | 3554-3618 | 65 | GAC |  |  | 0 |
| *ATP8* | + | 3619-3774 | 156 |  | ATT | TAG | 0 |
| *ATP6* | + | 3768-4442 | 675 |  | ATG | TAA | -1 |
| *COⅢ* | + | 4442-5228 | 787 |  | ATG | T | 0 |
| *tRNA^Gly^* | + | 5229-5293 | 65 | GGA |  |  | 0 |
| *ND3* | + | 5294-5647 | 354 |  | TTG | TAG | -2 |
| *tRNA^Ala^* | + | 5646-5711 | 66 | GCA |  |  | 0 |
| *tRNA^Arg^* | + | 5712-5776 | 65 | CGA |  |  | -2 |
| *tRNA^Asn^* | + | 5775-5838 | 64 | AAC |  |  | 0 |
| *tRNA^Ser1^* | + | 5839-5905 | 67 | AGA |  |  | 0 |
| *tRNA^Glu^* | + | 5906-5972 | 67 | GAA |  |  | -2 |
| *tRNA^Phe^* | - | 5971-6036 | 66 | TTC |  |  | 0 |
| *ND5* | - | 6037-7753 | 1717 |  | ATA | T | -3 |
| *tRNA^His^* | - | 7751-7818 | 68 | CAC |  |  | +5 |
| *ND4* | - | 7824-9158 | 1335 |  | ATG | TAA | -7 |
| *ND4L* | - | 9152-9439 | 288 |  | ATG | TAG | +18 |
| *tRNA^Thr^* | + | 9458-9525 | 68 | ACA |  |  | 0 |
| *tRNA^Pro^* | - | 9526-9589 | 64 | CCA |  |  | +2 |
| *ND6* | + | 9592-10098 | 507 |  | ATT | TAA | -1 |
| *Cyt b* | + | 10098-11237 | 1140 |  | ATG | TAA | -1 |
| *tRNA^Ser2^* | + | 11237-11304 | 68 | TCA |  |  | +17 |
| *ND1* | - | 11269-11322 | 948 |  | ATA | TAG | +4 |
| *tRNA^Leu1^* | - | 12274-12342 | 69 | CTA |  |  | 0 |
| *16S rRNA* | - | 12343-13618 | 1276 |  |  |  | 0 |
| *tRNA^Val^* | - | 13619-13688 | 70 | GTA |  |  |  |
| *12S rRNA* | - | 13689-14491 | (incomplete) |  |  |  |  |

**Table S1.** Location of features in the mtDNA of *Au. atronotatus*
