# Supplementary material for: Mitogenome Analysis of Four Lamiinae Species (Coleoptera: Cerambycidae) and Gene Expression Responses by Monochamus alternatus When Infected with the Parasitic Nematode, Bursaphelenchus mucronatus
Source: Insects. 2021 May 14;12(5):453. doi: 10.3390/insects12050453 (PMC8157225; doi:10.3390/insects12050453)
Supplement: Supplementary file 1 [file insects-12-00453-s001.zip › insects-1211092-supplementary/Supplementary Materials/Table S2 .docx]

| Gene | Strand | Position | Length  (nuc.) | Anticodon | Start  codon | Stop  codon | Intergenic  nucleotides |
| --- | --- | --- | --- | --- | --- | --- | --- |
| *tRNA^Ile^* | + | 1-68 | 68 | ATC |  |  | +9 |
| *tRNA^Gln^* | - | 78-146 | 69 | CAA |  |  | -1 |
| *tRNA^Met^* | + | 146-214 | 69 | ATG |  |  | 0 |
| *ND2* | + | 215-1225 | 1011 |  | ATT | TAA | -2 |
| *tRNA^Trp^* | + | 1224-1291 | 68 | TGA |  |  | -8 |
| *tRNA^Cys^* | - | 1284-1343 | 60 | TGC |  |  | 0 |
| *tRNA^Tyr^* | - | 1344-1409 | 66 | TAC |  |  | -8 |
| *COⅠ* | + | 1402-2949 | 1548 |  | ATT | TAA | -5 |
| *tRNA^Leu2^* | + | 2945-3010 | 66 | TTA |  |  | 0 |
| *COⅡ* | + | 3011-3698 | 688 |  | ATC | T | 0 |
| *tRNA^Lys^* | + | 3699-3767 | 69 | AAA |  |  | 0 |
| *tRNA^Asp^* | + | 3768-3835 | 68 | GAC |  |  | 0 |
| *ATP8* | + | 3836-3991 | 156 |  | ATT | TAA | -4 |
| *ATP6* | + | 3988-4659 | 672 |  | ATG | TAA | -1 |
| *COⅢ* | + | 4659-5447 | 789 |  | ATG | TAA | -1 |
| *tRNA^Gly^* | + | 5447-5511 | 65 | GGA |  |  | 0 |
| *ND3* | + | 5512-5865 | 354 |  | ATA | TAG | -2 |
| *tRNA^Ala^* | + | 5864-5927 | 64 | GCA |  |  | 0 |
| *tRNA^Arg^* | + | 5928-5991 | 64 | CGA |  |  | -1 |
| *tRNA^Asn^* | + | 5991-6055 | 65 | AAC |  |  | 0 |
| *tRNA^Ser1^* | + | 6056-6121 | 66 | AGA |  |  | 0 |
| *tRNA^Glu^* | + | 6122-6185 | 64 | GAA |  |  | -1 |
| *tRNA^Phe^* | - | 6185-6248 | 64 | TTC |  |  | 0 |
| *ND5* | - | 6249-7965 | 1717 |  | ATA | T | -3 |
| *tRNA^His^* | - | 7963-8026 | 64 | CAC |  |  | 0 |
| *ND4* | - | 8027-9359 | 1333 |  | ATG | T | -7 |
| *ND4L* | - | 9353-9640 | 288 |  | ATG | TAA | 2 |
| *tRNA^Thr^* | + | 9643-9707 | 65 | ACA |  |  | 0 |
| *tRNA^Pro^* | - | 9708-9772 | 65 | CCA |  |  | 2 |
| *ND6* | + | 9775-10278 | 504 |  | ATT | TAA | -1 |
| *Cyt b* | + | 10278-11417 | 1140 |  | ATG | TAA | -1 |
| *tRNA^Ser2^* | + | 11417-11484 | 68 | TCA |  |  | +24 |
| *ND1* | - | 11509-12456 | 948 |  | GTA | TAG | +4 |
| *tRNA^Leu1^* | - | 12461-12525 | 65 | CTA |  |  | 0 |
| *16S rRNA* | - | 12526-13809 | 1284 |  |  |  | 0 |
| *tRNA^Val^* | - | 13810-13880 | 71 | GTA |  |  | 0 |
| *12S rRNA* | - | 13881-14692 | 812 |  |  |  |  |
| CR |  | 14693-14858 | (incomplete) |  |  |  |  |

**Table S2.** Location of features in the mtDNA of *Ap. germari.*
