# Supplementary material for: Mitogenome Analysis of Four Lamiinae Species (Coleoptera: Cerambycidae) and Gene Expression Responses by Monochamus alternatus When Infected with the Parasitic Nematode, Bursaphelenchus mucronatus
Source: Insects. 2021 May 14;12(5):453. doi: 10.3390/insects12050453 (PMC8157225; doi:10.3390/insects12050453)
Supplement: Supplementary file 1 [file insects-12-00453-s001.zip › insects-1211092-supplementary/Supplementary Materials/Table S3.docx]

**Table S3.** Location of features in the mtDNA of *P. fortunei*

| Gene | Strand | Position | Length  (nuc.) | Anticodon | Start  codon | Stop  codon | Intergenic  nucleotides |
| --- | --- | --- | --- | --- | --- | --- | --- |
| *tRNA^Ile^* | + | 1-65 | 65 | ATC |  |  | -3 |
| *tRNA^Gln^* | - | 63-131 | 137 | CAA |  |  | -1 |
| *tRNA^Met^* | + | 131-199 | 69 | ATG |  |  | 0 |
| *ND2* | + | 200-1207 | 1008 |  | ATC | TAA | -2 |
| *tRNA^Trp^* | + | 1206-1271 | 66 | TGA |  |  | -8 |
| *tRNA^Cys^* | - | 1264-1326 | 63 | TGC |  |  | 0 |
| *tRNA^Tyr^* | - | 1327-1390 | 64 | TAC |  |  | -8 |
| *COⅠ* | + | 1383-2930 | 1548 |  | ATT | TAA | -5 |
| *tRNA^Leu2^* | + | 2926-2990 | 65 | TTA |  |  | 0 |
| *COⅡ* | + | 2991-3678 | 688 |  | ATT | T | 0 |
| *tRNA^Lys^* | + | 3679-3748 | 70 | AAA |  |  | +3 |
| *tRNA^Asp^* | + | 3752-3817 | 66 | GAC |  |  | 0 |
| *ATP8* | + | 3818-3973 | 156 |  | ATT | TAA | -7 |
| *ATP6* | + | 3967-4641 | 675 |  | ATG | TAA | -1 |
| *COⅢ* | + | 4641-5429 | 789 |  | ATG | TAA | +2 |
| *tRNA^Gly^* | + | 5432-5497 | 66 | GGA |  |  | 0 |
| *ND3* | + | 5498-5851 | 354 |  | ATT | TAG | -2 |
| *tRNA^Ala^* | + | 5850-5914 | 65 | GCA |  |  | 0 |
| *tRNA^Arg^* | + | 5915-5976 | 62 | CGA |  |  | -1 |
| *tRNA^Asn^* | + | 5976-6039 | 64 | AAC |  |  | 0 |
| *tRNA^Ser1^* | + | 6040-6106 | 67 | AGA |  |  | 0 |
| *tRNA^Glu^* | + | 6107-6169 | 63 | GAA |  |  | -1 |
| *tRNA^Phe^* | - | 6169-6233 | 65 | TTC |  |  | 0 |
| *ND5* | - | 6234-7947 | 1714 |  | ATG | T | +1 |
| *tRNA^His^* | - | 7949-8011 | 63 | CAC |  |  | 0 |
| *ND4* | - | 8012-9338 | 1327 |  | ATA | T | -4 |
| *ND4L* | - | 9335-9622 | 288 |  | ATG | TAA | +2 |
| *tRNA^Thr^* | + | 9625-9687 | 63 | ACA |  |  | 0 |
| *tRNA^Pro^* | - | 9688-9753 | 66 | CCA |  |  | +2 |
| *ND6* | + | 9756-10259 | 504 |  | ATT | TAA | -1 |
| *Cyt b* | + | 10259-11398 | 1140 |  | ATG | TAG | -2 |
| *tRNA^Ser2^* | + | 11397-11464 | 68 | TCA |  |  | -19 |
| *ND1* | - | 11484-12434 | 951 |  | TTG | TAA | +1 |
| *tRNA^Leu1^* | - | 12436-12498 | 63 | CTA |  |  | 0 |
| *16S rRNA* | - | 12499-13776 | 1278 |  |  |  | 0 |
| *tRNA^Val^* | - | 13777-13846 | 70 | GTA |  |  | -2 |
| *12S rRNA* | - | 13845-14626 | 782 |  |  |  | 0 |
| *CR* |  | 14627-15472 | 846 |  |  |  |  |
