# Supplementary material for: Mitogenome Analysis of Four Lamiinae Species (Coleoptera: Cerambycidae) and Gene Expression Responses by Monochamus alternatus When Infected with the Parasitic Nematode, Bursaphelenchus mucronatus
Source: Insects. 2021 May 14;12(5):453. doi: 10.3390/insects12050453 (PMC8157225; doi:10.3390/insects12050453)
Supplement: Supplementary file 1 [file insects-12-00453-s001.zip › insects-1211092-supplementary/Supplementary Materials/Table S4 .docx]

| Gene | Strand | Position | Length  (nuc.) | Anticodon | Start  codon | Stop  codon | Intergenic  nucleotides |
| --- | --- | --- | --- | --- | --- | --- | --- |
| *ND2* | + | 1-1011 | 1011 |  | ATT | TAA | -2 |
| *tRNA^Trp^* | + | 1010-1076 | 67 | TGA |  |  | -8 |
| *tRNA^Cys^* | - | 1069-1130 | 62 | TGC |  |  | 0 |
| *tRNA^Tyr^* | - | 1131-1196 | 66 | TAC |  |  | -8 |
| *COⅠ* | + | 1189-2731 | 1543 |  | ATT | T | 0 |
| *tRNA^Leu2^* | + | 2732-2796 | 65 | TTA |  |  | 0 |
| *COⅡ* | + | 2797-3483 | 687 |  | ATT | T | +1 |
| *tRNA^Lys^* | + | 3485-3554 | 70 | AAA |  |  | -1 |
| *tRNA^Asp^* | + | 3554-3620 | 67 | GAC |  |  | 0 |
| *ATP8* | + | 3621-3776 | 156 |  | ATT | TAA | -7 |
| *ATP6* | + | 3770-4444 | 675 |  | ATG | TAA | -1 |
| *COⅢ* | + | 4444-5230 | 787 |  | ATG | T | 0 |
| *tRNA^Gly^* | + | 5231-5295 | 65 | GGA |  |  | 0 |
| *ND3* | + | 5296-5649 | 354 |  | ATT | TAG | -2 |
| *tRNA^Ala^* | + | 5648-5712 | 65 | GCA |  |  | 0 |
| *tRNA^Arg^* | + | 5713-5775 | 63 | CGA |  |  | 0 |
| *tRNA^Asn^* | + | 5776-5843 | 68 | AAC |  |  | 0 |
| *tRNA^Ser1^* | + | 5844-5910 | 67 | AGA |  |  | 0 |
| *tRNA^Glu^* | + | 5911-5973 | 63 | GAA |  |  | -1 |
| *tRNA^Phe^* | - | 5973-6039 | 67 | TTC |  |  | 0 |
| *ND5* | - | 6040-7753 | 1714 |  | ATT | T | 0 |
| *tRNA^His^* | - | 7754-7817 | 64 | CAC |  |  | 0 |
| *ND4* | - | 7818-9150 | 1333 |  | ATG | T | -7 |
| *ND4L* | - | 9144-9431 | 288 |  | ATG | TAA | +2 |
| *tRNA^Thr^* | + | 9434-9496 | 63 | ACA |  |  | 0 |
| *tRNA^Pro^* | - | 9497-9560 | 64 | CCA |  |  | +2 |
| *ND6* | + | 9563-10066 | 504 |  | ATT | TAA | -1 |
| *Cyt b* | + | 10066-11205 | 1140 |  | ATG | TAG | -2 |
| *tRNA^Ser2^* | + | 11204-11269 | 66 | TCA |  |  | +17 |
| *ND1* | - | 11287-12234 | 948 |  | ATA | TAG | +4 |
| *tRNA^Leu1^* | - | 12239-12303 | 65 | CTA |  |  | -1 |
| *16S rRNA* | - | 12303-13572 | 1270 |  |  |  | 0 |
| *tRNA^Val^* | - | 13573-13641 | 69 | GTA |  |  |  |
| *12S rRNA* | - | 13642-14189 | (incomplete) |  |  |  |  |

**Table S4.** Location of features in the mtDNA of *M. alternatus.*
