# Supplementary figures and images for: Mitogenome Analysis of Four Lamiinae Species (Coleoptera: Cerambycidae) and Gene Expression Responses by Monochamus alternatus When Infected with the Parasitic Nematode, Bursaphelenchus mucronatus
Source: Insects. 2021 May 14;12(5):453. doi: 10.3390/insects12050453 (PMC8157225; doi:10.3390/insects12050453)

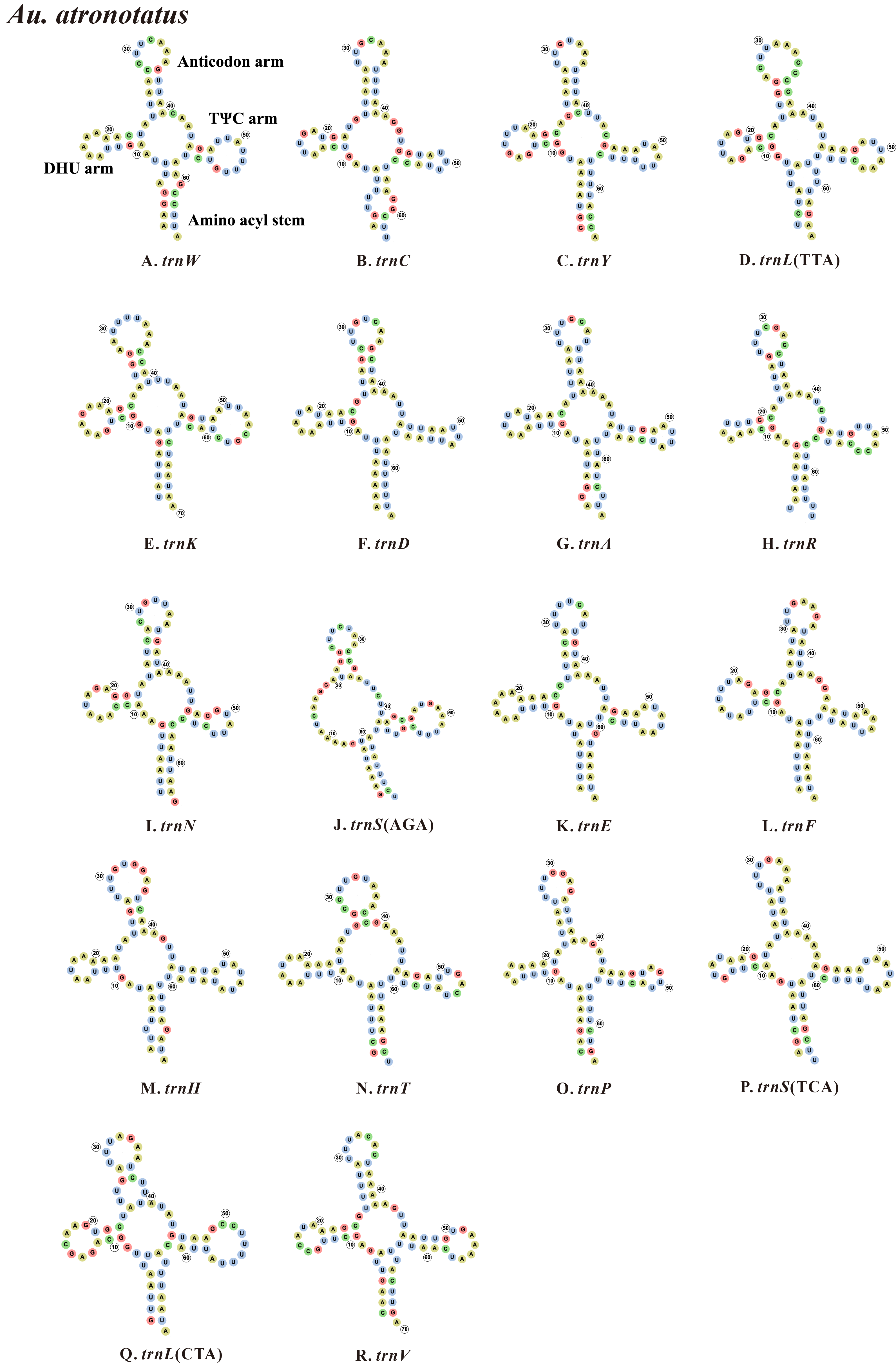

Supplement: Supplementary file 1 [file insects-12-00453-s001.zip › insects-1211092-supplementary/Supplementary Materials/Figure S1.png]

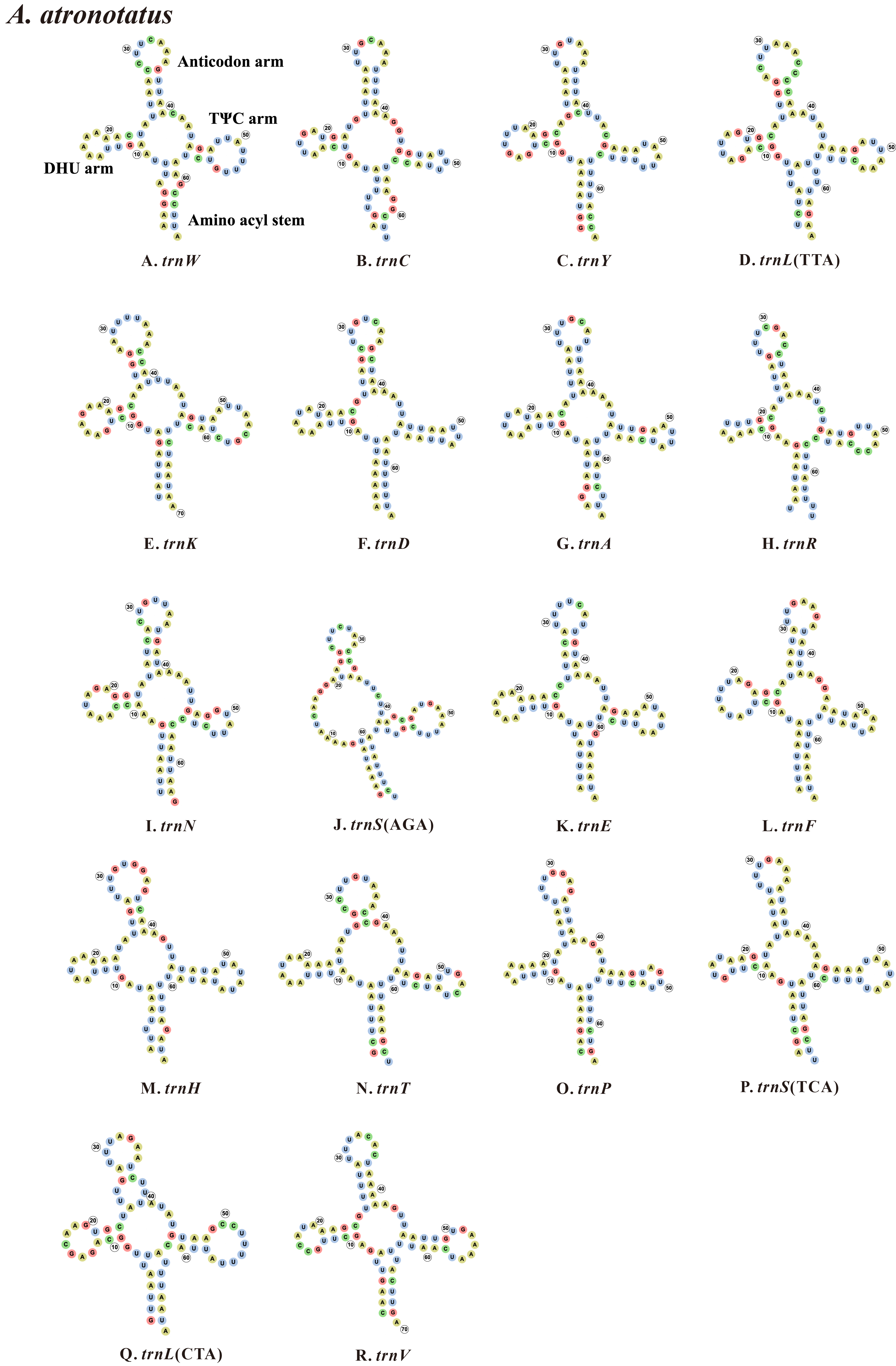

Supplement: Supplementary file 1 [file insects-12-00453-s001.zip › insects-1211092-supplementary/Supplementary Materials/Figure S2.png]

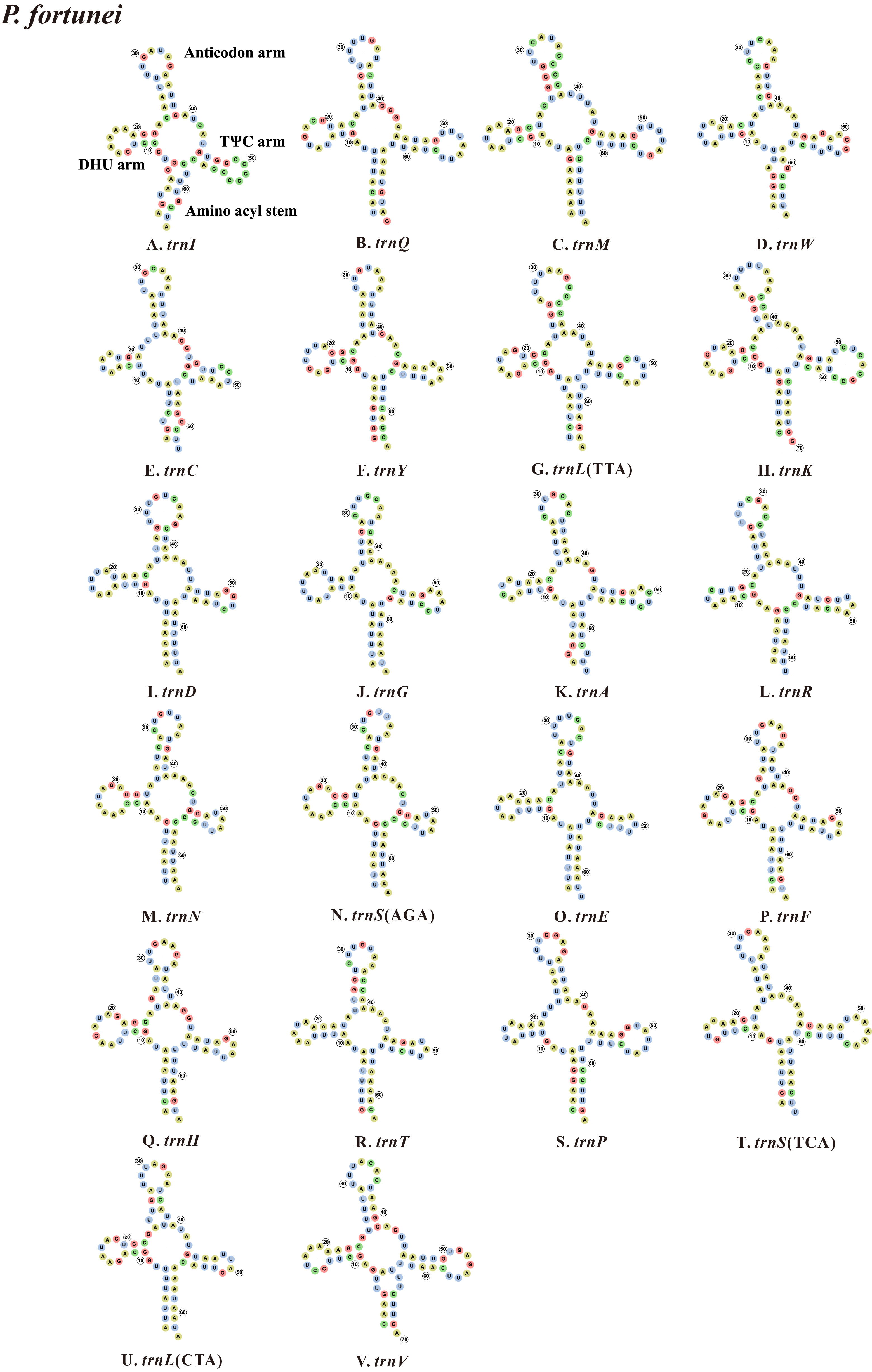

Supplement: Supplementary file 1 [file insects-12-00453-s001.zip › insects-1211092-supplementary/Supplementary Materials/Figure S3.png]

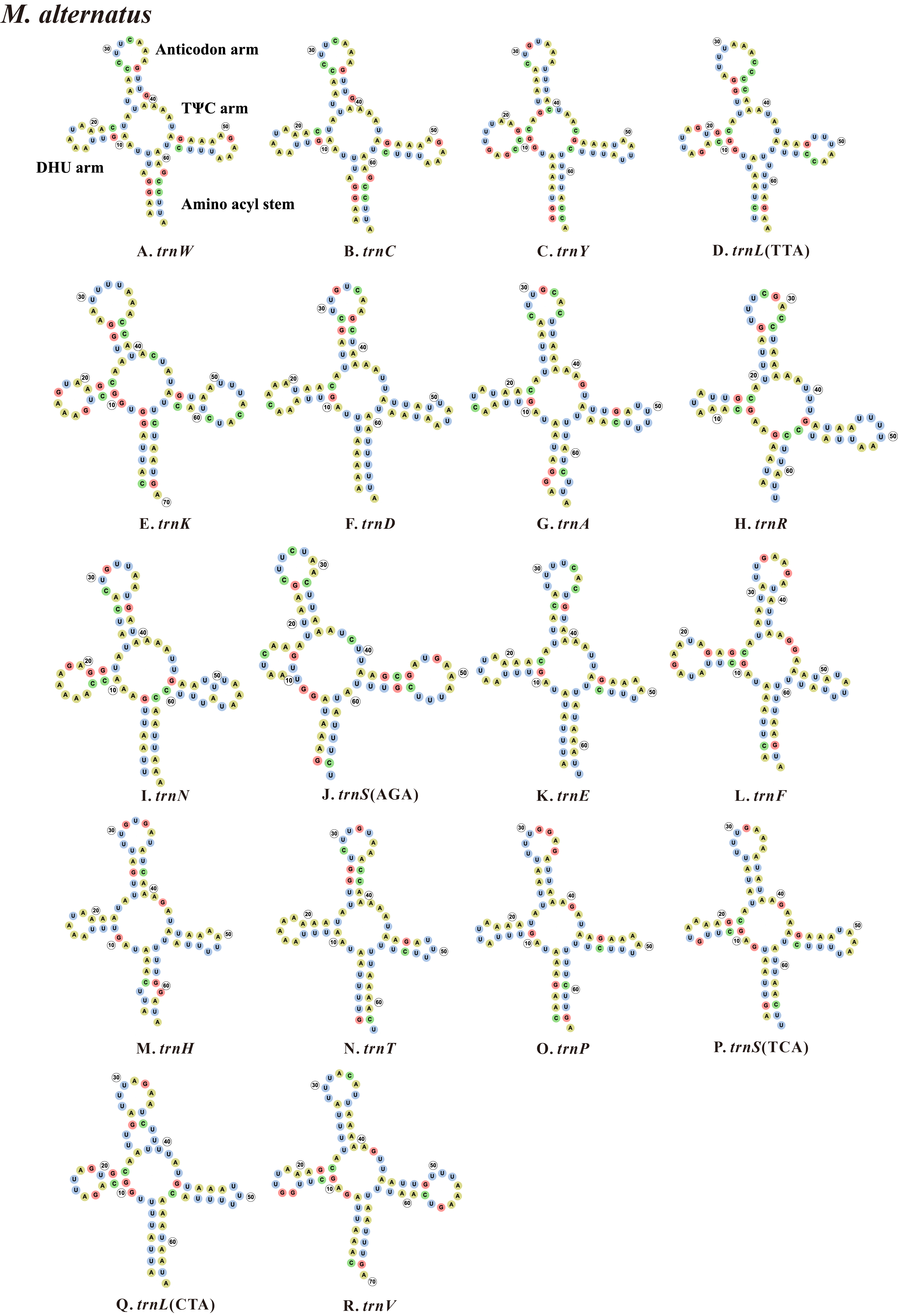

Supplement: Supplementary file 1 [file insects-12-00453-s001.zip › insects-1211092-supplementary/Supplementary Materials/Figure S4.png]
